# In search of the relationship between the rye polyamine oxidase (PAO) gene and resistance to powdery mildew (PM)

**DOI:** 10.1007/s13353-022-00723-x

**Published:** 2022-09-30

**Authors:** Paweł Milczarski, Magdalena Góralska, Kinga Pałatyńska, Bartłomiej Wysoczański, Ilona Czyczyło-Mysza, Fatemeh Maghuly, Beata Myśków

**Affiliations:** 1 Department of Plant Genetics, Breeding and Biotechnology, West-Pomeranian University of Technology (ZUT), ul. Słowackiego 17, 71–434, Szczecin, Poland; 2 HYBRO Saatzucht GmbH & Co. KG, Station Kleptow, Kleptow 53, 17291 Schenkenberg, Germany; 3 Polish Academy of Sciences, The Franciszek Gorski Institute of Plant Physiology, Niezapominajek 21, 30–239, Kraków, Poland; 4 Plant Function Genomic, Institute of Molecular Biotechnology, Department Biotechnology, University of Natural Resources and Life Sciences, Muthgasse 18, 1190 Vienna (BOKU), Austria

**Keywords:** Biotic stress, *Erysiphe graminis*, Genetic mapping, QTL, Fungus resistance, *Secale cereale* L

## Abstract

Powdery mildew (PM), a common cereal disease in cultivated areas, including Europe and other temperate regions, is caused by the fungus *Blumeria graminis*. While PM is one of the most important wheat leaf diseases globally, rye is highly tolerant to PM. It has been reported that in barley infected with PM, polyamine oxidase (PAO) activity related to the production of hydrogen peroxide (H_2_O_2_) has increased, which may promote defense against biotrophic or hemibiotrophic pathogens. The current study aimed to assess the relationship between the segregation of the polymorphic marker for rye *PAO* (*ScPAO*) and the level of PM infection in plants. The genetic mapping in two interline populations shows that *ScPAO* is located on chromosome 7R. Further analysis comparing *ScPAO* location to mapped wheat (*Triticum aestivum* L.) *PAO* duplicates suggests the *ScPAO* homology with *TaPAO6* or *TaPAO7*. A possible association of *ScPAO* from 7R with PM resistance is demonstrated in the recombinant inbred lines (RIL)-L population phenotyped for PM infection. Finally, three novel QTLs for PM resistance on the 7R chromosome of rye are detected.

**Supplementary Information:**

The online version contains supplementary material available at 10.1007/s13353-022-00723-x.

## Introduction 

Rye (*Secale cereale* L.) is a cultivated plant of great agronomic importance. Along with wheat, barley, and oats, rye is one of the main crops grown in Poland and other European countries with a temperate climate, although to a lesser extent, rye is also grown in Canada, the northern USA, Japan, Australia and the Republic of South Africa. From the economic point of view, the most important features of rye are good tolerance to low temperatures and other stresses, low soil requirements, and thus also low requirements for the use of fertilizers and pesticides. The size of the rye cultivation area in the world results from its versatile use in baking, fodder, brewing, and distilling industries. In 2020, the area of rye cultivation in Europe accounted for 81% of the total rye cultivated area (3.6 × 10^6^ and 4.4 × 10^6^ ha, respectively), while the rye cultivation area in Poland accounted for 23% (8.4 × 10^5^ ha) of the European crops. Rye production in 2020 was correlated with the cultivated area, so European production accounted for 86% of world production, 1.2 × 10^10^ and 1.5 × 10^10^ kg, respectively, while Polish production accounted for 22% (2.9 × 10^9^ kg) of the European production (www.fao.org/faostat).

Rye is diploid (2n = 14), with seven chromosomes designated as 1R–7R, closely related to wheat (*Triticum aestivum* L.), indicated by the partial homology of the chromosomes of both rye and wheat (Crespo-Herrera et al. 2017; Devos et al. 1993; Li et al. 2013). Rye has the largest haploid genome of the *Triticeae*, with a genome size of approximately 7.9 Gbp, of which over 90% are repetitive sequences (Bauer et al. 2017). The first attempt to sequence the rye genome was made by Bauer et al. (2017), achieving 35% genome coverage. The work of two teams of scientists on whole-genome sequencing projects were completed four years later (Li et al. 2021; Rabanus-Wallace et al. 2021).

Due to the exceptional resistance of rye to abiotic stresses, it is used as a donor of resistance genes for the less resilient wheat (Crespo-Herrera et al. 2017), which makes it possible to grow this grain in cooler climates.

PAOs play an important role in the metabolism of polyamines and are involved in several physiological processes by regulating the levels of polyamines and reaction products. PAOs are characterized by high variability of substrate specificity, catalytic mechanism, and subcellular localization, and they influence key cellular processes in various organisms (Tavladoraki et al. 2006; Salvi and Tavladoraki 2020). Putrescine (Put), spermidine (Spd), and spermine (Spm) are the main polyamines (PAs) in plants, and they play a key role in several developmental processes. They are involved in responses to various types of abiotic stress (such as potassium deficiency, osmotic shock, drought, and salt stress) and plant-pathogen interactions (Tavladoraki et al. 2006). It was reported that PAO activities connected with H_2_O_2_ production might play a role in promoting defense against biotrophic or hemibiotrophic pathogens (reviewed in: Chen et al. 2019; reviewed in: Wang et al. 2019). Increased activity of diamine and polyamine oxidases was demonstrated in barley infected with the obligatory biotroph of *Blumeria graminis*, causing PM in cereals (Asthir et al. 2004; Cowley and Walters 2002).

PM is a widespread cereal disease in cultivation areas, including Europe and other temperate regions (Asthir et al. 2004). While PM is one of wheat’s most important foliar diseases, rye is highly tolerant to PM. Therefore, rye is a widely used source of resistance genes for wheat improvement. Over ten monogenic *Pm* genes conferring resistance to PM were identified in the rye (reviewed in: Rakoczy-Trojanowska et al. 2021). At least three wheat *Pm* resistance genes are derived from rye (*Pm*7, *Pm*8/*Pm*17, *Pm*20) (reviewed in: Hao et al. 2018). As the monogenic disease resistance is not permanent and is often overcome by pathogens (Han et al. 2022), it is important to thoroughly understand the various resistance mechanisms, including those with a polygenic background.

In the current study, the research aimed to analyze PAO transcripts of winter rye of selected inbred lines, to develop a PCR marker for *PAO*, to determine the location of the gene on the genetic map of rye, to assess the correlation between *PAO* allele segregation and PM infection and to search for QTLs for PM resistance.

## Materials and methods

Transcriptomic data (Bienias et al. 2020) archived at the National Center for Biotechnology Information (NCBI) under accession numbers from SRX2636904 to SRX2636920, under BioProject PRJNA371298, ID: 371,298 (http://www.ncbi.nlm.nih.gov/sra) was used to search for rye *PAO* genes. Primers were designed using Primer3 (https://www.ncbi.nlm.nih.gov/tools/primer-blast/).

Twelve inbred rye lines (541, Ot1-3, DS2, RXL10, S120, S76, M12, 08/LM, 2050, S32N/07, AK1-2, AKZ) of various origins were used for detecting the polymorphic markers. In addition, two mapping populations of recombinant inbred lines (RIL), RIL-K and RIL-L (Milczarski et al. 2011), were used for *ScPAO* mapping.

*ScPAO* mapping was performed using the dense genetic maps constructed with DArT (diversity array technology) markers (Milczarski et al. 2011). Only DArTs with known sequences and confirmed physical localizations were used for remapping 7R with *ScPAO* segregation. The set of selected DArTs with known sequences from the RIL-K and RIL-L populations were mapped to the reference whole genome sequence of rye (Rabanus-Wallace et al. 2021) using Geneious 11.1 software, with the default recommended sensitivity and Geneious assembler with Q30 mapping level. Genetic mapping was performed using the JoinMap 5.0 computer program. QTL mapping was performed with the software package WinQTL Cartographer 2.5 using the composite interval mapping (CIM) procedure, with the threshold value of LOD set at a score described by the permutation test (500 permutation times at significance level *P* = 0.05). MapChart 2.32 software was used to draw the genetic maps.

The phenotypic analysis of PM infection was performed over two different years (2005, 2006), in two replicates each year, using the RIL-L population. Each genotype was represented by 3–5 biological replication grown in an experimental field (in 80 cm long rows separated by 17 cm) and greenhouse (in pots filled with the soil mass of 3.8 kg) of the ZUT. Phenotyping was performed four times; the symbols PM05a, PM05b, PM06a, and PM06b represent repetitions of experiments (the data were obtained in 2005 from the field, and the greenhouse, and in 2006 from the field and the greenhouse, respectively). The experiment was conducted without artificial inoculation and without fungicide spraying. Infection was assessed using a 9-point scale, where 1 refers to the most severe infection and 9 to no signs of infection.

Statistical tests for significance of differences (*t*-test and non-parametric *U* Mann–Whitney test) were performed using STATISTICA v13.0 (www.statsoft.com).

## Results and discussion

The idea behind the current study is to locate the PAO gene on rye’s genetic map and evaluate its relationship with PM. The *PAOs* were best characterized in *Arabidopsis thaliana*, where five different cDNA fragments (*AtPAO1*-*AtPAO5*) encoding putative PAOs were identified. These enzymes exhibit different subcellular localization, substrate specificity, and functional diversity (Tavladoraki et al. 2006; reviewed in: Wang et al. 2019). Analysis of the rye transcriptome of four near-isogenic lines (NIL) pairs (Bienias et al. 2020) showed the presence of the *ScPAO1*-*ScPAO5* and *ScPAOX* (peroxisomal N1-acetyl-Spm/Spd oxidase) transcripts. *ScPAO1* (1848 nt transcript) was selected for further study as the only one identified in two of four NILs (the remaining homologues were detected in all four lines). Gene ontology (GO) analysis indicated *Arabidopsis AtPAO1*, while NCBI BLAST analysis identified *TaPAO6*, *TaPAO1*, and *TaPAO7* of wheat as the most similar sequences to this transcript (total score: 2,766; 2,584; 2,514; respectively). Since the identity of *ScPAO* could not be established, the gene has not been assigned a number in the next part of the study.

**Fig. 1:**
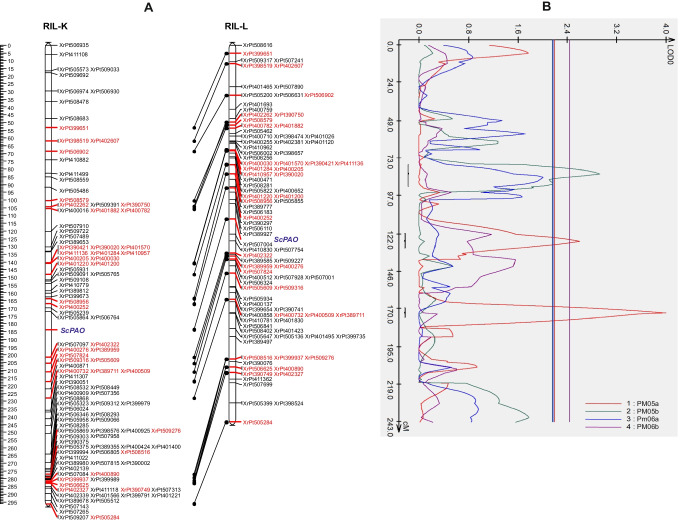
**A** The position of *ScPAO* on the genetic map of rye chromosome 7R of two mapping populations (RIL-K and RIL-L) and **B** QTLs for PM resistance on the map RIL-L. The PM05a, PM05b, PM06a, and PM06b represent repetitions of phenotypic experiments (the obtained phenotypes in 2005 from the field and the greenhouse and in 2006 from the field and the greenhouse, respectively)

Out of six one primer pair (F: TTGACGATGCGAGGATACTG, R: CTTCAAAGTCCGTCCCAAGT) was selected to show interline polymorphism. The length of the amplified *ScPAO* fragment (approx. 170nt) was longer than that predicted from the transcript sequence (97nt), suggesting the presence of an intron sequence. This polymorphic marker was amplified in three of 12 inbred lines. The amplicon presence in lines 541 and RXL10, allowed for linkage analysis in two immortal populations (RIL-K and RIL-L). In both cases, *ScPAO* was assigned to 7R (Fig. 1).

De novo–constructed genetics maps of 7R in RIL-K and RIL-L consists of 98 and 116 DArTs. Genetic marker segregations (DArT and *ScPAO* marker sequences) are reported in supplementary material (SMTab.1). The use of only physically verified markers resulted in an approximately consensus marker order on the genetic and physical maps of 7R with minor rearrangements (SM Fig. 1).

**Fig. 2:**
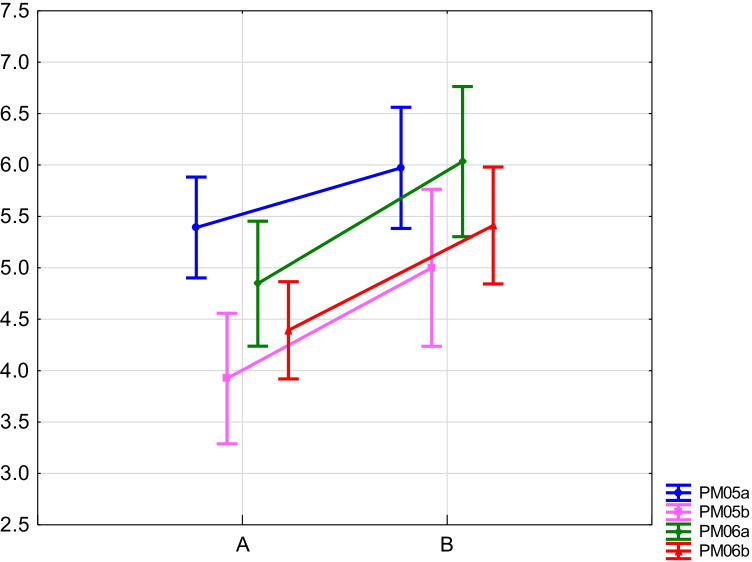
The level of PM infection (scale 1–9) in two groups of plants of RIL-L population divided according to opposite alleles of PCR marker for *ScPAO*; **A** allele from line DS2, **B** allele from line RXL10. Vertical bars represent ± standard error

The RIL-L mapping population was assessed for PM infection. Both parents, DS2 and RXL10, had a similar level of susceptibility (1 or 3 points of 9-point scale, depending on replicate), and the trait segregation in RIL-L ranged from 1 to 9 points, suggesting the transgression. Therefore, plants were conventionally divided into two groups in terms of polymorphism of PCR markers for the *ScPAO*: one with the “a” allele derived from maternal line DS2 (without amplicon), the other with the “b” allele—from parental line RXL10 (with amplicon). Comparison of the PM infections level in these two groups of plants, using both the *t*-test and the *U* Mann–Whitney test, showed a statistically significant difference for PM06b (*P* = 0.04); however, the trend was the same for all 4 replicates (Fig. 2). This result may indicate a potential association of *ScPAO* with resistance to PM. Furthermore, the CIM and de novo–constructed genetic map were used to search for an association between *ScPAO* and PM resistance. Three QTLs were identified on chromosome 7R of the RIL-L population, with maximum peaks located at positions 83.2 (LOD = 2.9, a =  − 1.94), 126.4 (LOD = 2.6, a =  + 0.88) and 172.9 cM (LOD = 4.0, a =  − 1.16). *ScPAO* locus was not within any of the QTL intervals but was close to that with a peak at 126.4 cM (Fig. 1). The stronger effect of nearby QTL may have prevented the detection of *ScPAO* as the QTL for PM resistance using the CIM method. Failure to detect QTL for PM in the vicinity of *ScPAO* does not preclude *ScPAO* from 7R from being associated with a response to PM infection, but may be related to its poor phenotypic effect (0.49–1.31, depending on replicate). The possible association of *ScPAO* with the response to PM confirms the obtained results with barley (Asthir et al. 2004; Cowley and Walters 2002). Barley researchers have suggested that the relationship between PAO and the response to PM infection is probably related to H_2_O_2_ production during an oxidative burst following an attack by the pathogen. The production of H_2_O_2_ is mediated by several enzymes on the surface of plant cells, e.g., PAOs. PAOs were shown to be involved in H_2_O_2_ production in developing barley grain (Asthir et al. 2004). Therefore, it may be that some of the H_2_O_2_ produced as a result of infection are a consequence of increased PAO activity.

At least 11 *Pm* resistance genes located on 1RS, 2RL, 3RS, 4R, 5RL, and 6RL were detected for rye (reviewed in: Rakoczy-Trojanowska et al. 2021). This number increases to 23 if we consider the genes found in the wheat-rye translocation lines, with rye being the donor parent (Vendelbo et al. 2021). The new genetic stocks of rye are still being sought to improve wheat. Recent studies report finding new YT2 germplasm with the 6RL *Pm* gene (Han et al. 2022).

Several *Pm* loci were identified in wheat chromosomal segments of 2A/B/D syntenic to rye chromosome arm 7RL (Ren et al. 2020). Until the recent detection of the *PmNOS1* PM resistance locus on the distal arm of chromosome 7RL (Vendelbo et al. 2021), no PM resistance gene was directly identified on rye chromosome 7R. Our most robust putative QTL located on the 7RL is possibly not identical to *PmNOS1* as it is not mapped to the distal tip of the chromosome. Thus, we could conclude that all three putative QTLs identified in the current study are novel (Fig. 1B).

Until now, PAO genes were not located on the genetic map of rye. The tested transcript showed significant identity to *TaPAO7*, *TaPAO6*, and *TaPAO1* (96.23%, 96.07%, and 94.84%, respectively). The analysis of the chromosomal location of the wheat *TaPAOs* (Gholizadeh and Mirzaghaderi 2020) could not clearly answer the question about the identity of *ScPAO*. The genetic map of chromosome 7R showed regions with homology to wheat groups 2, 4, 5, and 7 (Devos et al. 1993). The pericentromeric region of 7R, where *ScPAO* is located, corresponds to 4L and 7L (Devos et al. 1993; Li et al. 2013). The wheat group 4L carries the genes *TaPAO7* and *TaPAO10*, while 7L-*TaPAO6* and *TaPAO11* (Gholizadeh and Mirzaghaderi 2020). As *TaPAO1* is located on wheat chromosomes of group 3, *ScPAO* from 7R is probably homologic to *TaPAO6* or *TaPAO7*. The elucidation of which of the known *PAO* genes is rye homolog requires further structural or/and functional studies.

Although the relationship between biotic stress in various plants was reported (reviewed in: Chen et al 2019; reviewed in: Wang et al. 2019), the connection between PAO and PM has so far only been reported for barley (Asthir et al. 2004; Cowley and Walters 2002). Our research shows that *ScPAO* from 7R may be a gene involved in response to PM, of quantitative character and little additive effect, which was not recorded in the literature on the species and is probably not identical to any of the known PM genes of rye.

## Supplementary Information

Below is the link to the electronic supplementary material.

Supplementary file1 (PDF 46 KB)

Supplementary file2 (XLSX 136 KB)
