# Supplementary figures and images for: In search of the relationship between the rye polyamine oxidase (PAO) gene and resistance to powdery mildew (PM)
Source: J Appl Genet. 2022 Sep 30;64(1):65–70. doi: 10.1007/s13353-022-00723-x (PMC9836972; doi:10.1007/s13353-022-00723-x)

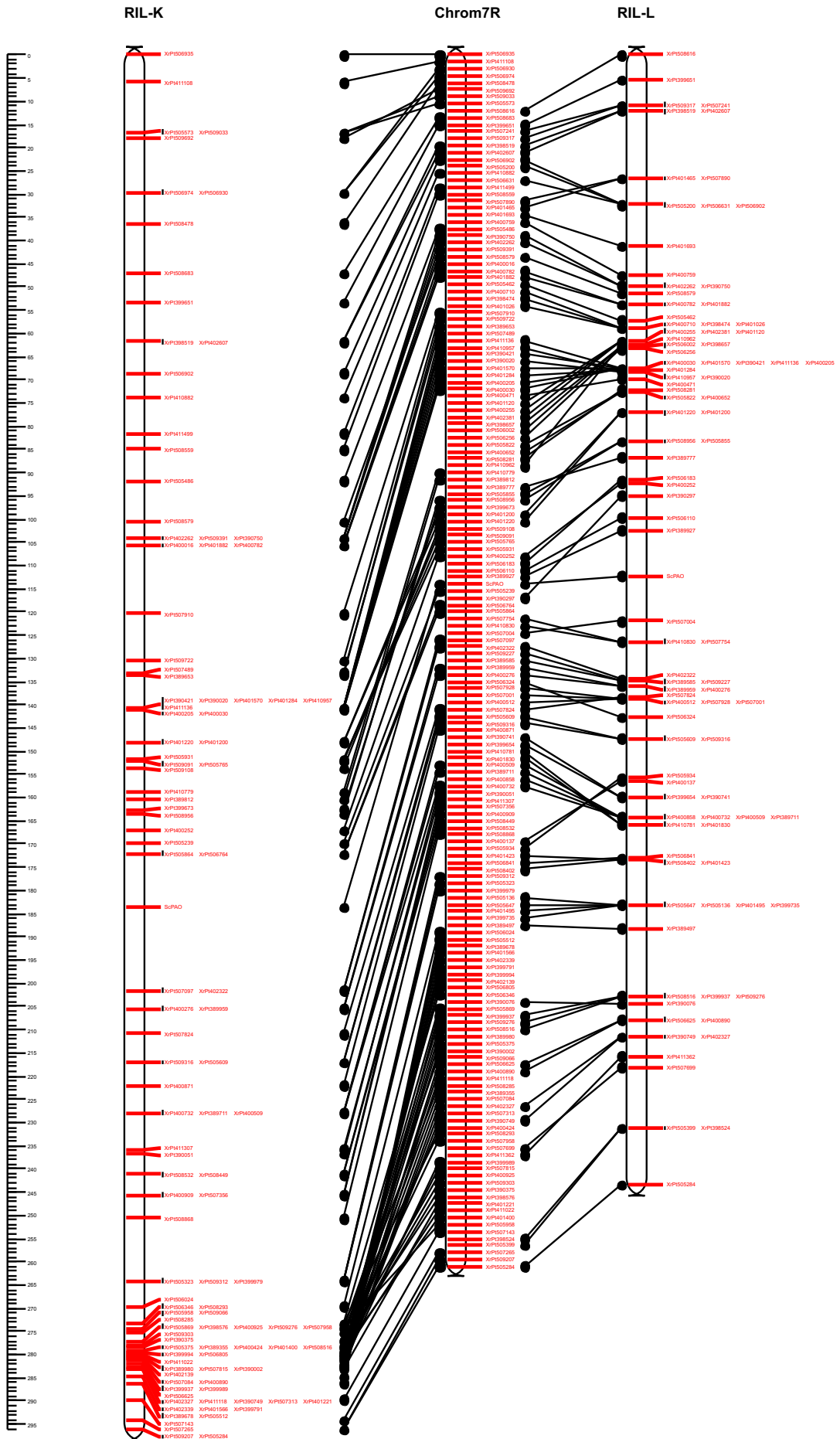

Supplement: Supplementary file 1 — Supplementary file1 (PDF 46 KB) [file 13353_2022_723_MOESM1_ESM.pdf]
